# Assessment of Genotoxic Biomarker in Tongue and Buccal Mucosal Epithelial Cells of COVID-19 Patients: An Observational Study

**DOI:** 10.7759/cureus.48706

**Published:** 2023-11-12

**Authors:** Jafer Sadik, Manish Sharma, Chetan V Deshmukh, Sameena B Maqhbool, Altaf H Thekiya, Priyanka P Kamble

**Affiliations:** 1 Department of Dentistry, Government Hospital, Periyur, IND; 2 Department of Oral Pathology, Jawahar Medical Foundation (JMF) Annasaheb Chudaman Patil Memorial (ACPM) Dental College, Dhule, IND; 3 Department of Public Health Dentistry, Nair Hospital Dental College, Mumbai, IND; 4 Department of Orthodontics, The Oxford Dental College, Bangalore, IND; 5 Department of Orthodontics and Dentofacial Orthopedics, Diamond Dental Care, Nanded, IND

**Keywords:** corona virus, genotoxicity, oral mucosa, feulgen reaction, micronuclei

## Abstract

Introduction: Angiotensin-converting enzyme 2 (ACE2) is the main host cell receptor for coronavirus disease 2019 (COVID-19) and is highly expressed in the tongue and buccal mucosa. Therefore, the present study was conducted to investigate genotoxic changes in epithelial cells of the buccal and tongue mucosa following COVID-19 infection.

Materials and methods: This study included 40 patients aged 25-40 years, divided into two groups: Group 1 (control group) included 20 healthy individuals with no prior history of COVID-19 infection subdivided into Group 1a (buccal mucosa), and Group 1b (tongue mucosa); Group 2 (case group) included 20 patients with a history of mild to moderate COVID-19 infection subdivided into Group 2a (buccal mucosa) and Group 2b (tongue mucosa). Genotoxic biomarkers, such as the number of micronuclei, pyknosis, karyolysis, and karyorrhexis, were assessed in epithelial cells from the buccal mucosa and the ventral surface of the tongue. Analysis of variance was used for intragroup comparisons, followed by post-hoc analysis using Tukey’s test.

Results: The mean age of the patients was 27.4±6.52 years. Statistically significant differences were observed between cases and controls in the number of micronuclei, pyknosis, karyolysis, and karyorrhexis in the epithelial cells of the buccal and tongue mucosa (p = 0.05).

Conclusion: SARS-CoV-2 has pronounced genotoxic effects on the epithelium of the ventral surface of the tongue in comparison to the buccal mucosa Therefore, patients with COVID-19 should be monitored regularly to develop future carcinomas, particularly those with habits of smoking, alcohol consumption, and tobacco usage.

## Introduction

Pandemically, COVID-19, also known as Coronavirus Disease-2019, has given rise to unparalleled worldwide consequences of SARS-CoV-2, otherwise referred to as severe acute respiratory syndrome coronavirus 2 [1]. Although transmission of SARS-CoV-2 becomes apparent through activities involving the oral cavity, such as speaking, breathing, coughing, and sneezing, the majority of attention has been directed toward the nasal-lung pathway of infection. Manifestations within the oral realm, including taste loss, dry mouth, and oral lesions, have been observed in approximately 50% of COVID-19 cases. However, it remains uncertain whether SARS-CoV-2 can directly invade and reproduce within oral tissues such as the salivary glands or mucosa [2]. The utilization of host entry factors, including angiotensin-converting enzyme 2 (ACE2) and members of the transmembrane protease serine (TMPRSS family, specifically TMPRSS2 and TMPRSS4), is employed by SARS-CoV-2 for its infection process [3]. To ascertain the vulnerabilities to infection across various body regions, it is crucial to understand the specific cell types that harbor these receptors [3]. The oral cavity is lined by stratified squamous epithelium, either keratinized or non-keratinized based upon site specificity. Owing to the heterogeneity in oral tissues at the cellular level, the effects of SARS-CoV-2 infection across various oral sites will also differ [4]. ACE2 and TMPRSS are commonly expressed in the oral epithelium; therefore, SARS CoV-2 has a tendency to affect the oral epithelial cells and produce genotoxic changes [2].

Micronuclei (MN), also referred to as genotoxic biomarkers, are additional cytoplasmic bodies located outside the nucleus and are formulated within cells by various genotoxic agents that cause damage to the chromosomes. The frequency at which MN appear serves as an indicator of chromosome breakage during the initial stages of cell division, and it is recognized that the quantity of MN increases in response to carcinogenic stimuli long before the manifestation of clinical symptoms [5]. Because over 90% of all malignancies in the human body originate from epithelial cells, the utilization of the MN assay using epithelial cells can serve as a viable method for monitoring and detecting elevated cancer susceptibility in the human population [6]. To date, exceptional paucity of studies to evaluate genotoxic damage in buccal mucosal cells of COVID-19 patients by micronucleus assay [7], and it was concluded that SARS-CoV-2 could induce mutagenesis and genotoxicity in oral cells. However, no study has been conducted to assess the genotoxic damage caused by SARS-CoV-2 in site-specific oral epithelia. Therefore, the primary objective of the present study was to perform a comparative analysis of genotoxic biomarkers in epithelial cells of buccal and tongue mucosa of patients with and without COVID-19.

## Materials and methods

An observational, prospective, cross-sectional study was conducted in the Department of Oral Pathology from October 2022 to August 2023. The study was approved by the institutional ethics committee (EC/NEW/INST/2022/2959/096) and adhered to the principles outlined in the Declaration of Helsinki. Furthermore, the study adhered to the Strengthening the Reporting of Observational Studies (STROBE) guidelines [8]. Written informed consent was obtained from all participants prior to the commencement of the study. Furthermore, any form of data revealing the identities of the subjects was kept confidential.

Sample size calculation

The sample size was calculated using GPOWER software (version 3.1, Franz Faul University of Kiel, Germany). The sample size was determined by taking into account the standard deviation of 0.5 and 1.5, which were obtained from a previous study for controls and cases, respectively [7]. The effect size used was 0.5. The power of the study was set at 90% with an alpha error of 5%. The estimated sample size was 20 patients per group.

Study design and eligibility criteria

A total of 220 patients were screened to select the 40 patients who were explained in detail about the study and provided informed consent to participate. Twenty patients with a history of COVID-19 in the past year and were diagnosed with COVID-19, based on real-time reverse transcription-polymerase chain reaction (RT-PCR) and laboratory reports, including plasma levels of white blood cells (WBCs), platelets, C-reactive protein (CRP), aspartate aminotransferase (AST), alanine aminotransferase (ALT), γ-glutamyltranspeptidase (GGT), alkaline phosphatase, and lactate dehydrogenase (LDH). The control group consisted of 20 healthy patients who did not have any history of COVID-19 infection.

Inclusion and exclusion criteria

Subjects aged 20-40 years, both males and females, who gave their consent to participate, and who had not taken any systemic antibiotics or antifungal drugs in the past six months, were included in the study. Patients who had systemic conditions affecting the oral cavity, lesions or radiation exposure of the oral cavity, pregnant or lactating females, patients on immunosuppressants, subjects who had taken anti-fungal drugs or steroids or antiviral drugs in the past six months, smokers, and alcoholics were excluded from the study. The inclusion criterion for the test group was a positive history of COVID-19 in the past one year. All patients in the COVID-19 group had mild to moderate COVID-19 and received symptomatic treatment at home for fever using antipyretics, maintenance of hydration, steam inhalation, warm saline rinses, and regular monitoring of temperature and saturation of peripheral oxygen (SpO2) levels. Detailed data were obtained, including demographics, medical records comprising laboratory tests, RT-PCR reports, clinical findings, radiological findings based on computed tomography scans or chest X-rays, and the course of treatment offered. In addition, their dental history was recorded in detail, including the last dental visit, oral hygiene methods, and type of diet. All participants were asked about the presence of symptoms of COVID-19, such as fever, cough, shortness of breath, headache, and fatigue, at present or in the past month. None of the patients reported any COVID-19 symptoms.

Group allocation and study procedure

40 patients were equally divided into two groups as follows: Group 1, control group with healthy subjects further divided into subgroups of buccal (Group 1a) and tongue mucosa (Group 1b); and Group 2, case group with subjects with a positive history of COVID-19 infection further divided into subgroups of buccal (Group 2a) and tongue mucosa (Group 2b). Samples were obtained from the buccal and tongue mucosa of all the subjects.

Collection of samples

**Figure 1:**
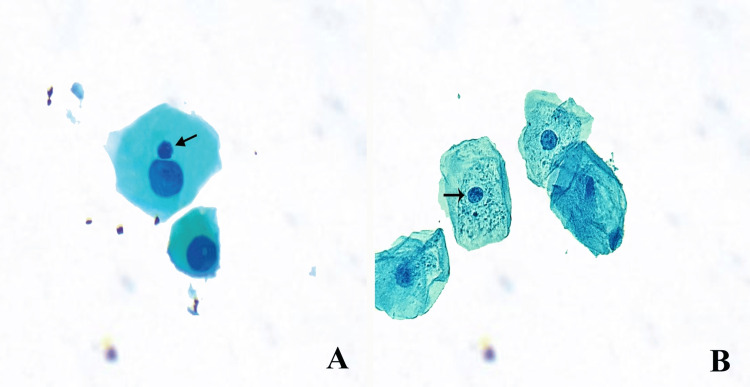
Epithelial cells stained with feulgen stain at 40x magnification. (A) Micronuclei (arrowhead) and (B) Pyknosis (arrowhead)

Prior to sample procurement, the participants underwent thorough rinsing of the oral cavity with water to eradicate undesirable waste matter. Buccal epithelial cells were collected by delicately scraping the inner linings of both the right and left cheek mucosa using a sterile wooden spatula that had been pre-moistened. As for the acquisition of tongue mucosal cells, they were obtained from the ventral surface of the tongue. Subsequently, cells derived from the mucosa were transferred into prelabelled Falcon tubes containing saline solution. Subsequently, the cells were centrifuged at 1,000 revolutions per minute for 5 min. The cells were fixed using a mixture of methanol and acetic acid at a ratio of 3:1. Finally, the fixed cells were evenly distributed across glass slides. All prepared slides were stained using the Feulgen/Fast Green method as described in a previously published protocol [9]. Cytocentrifugation of mucosal cells represents advancement in comparison to direct smear because of its ability to yield a uniform distribution and evenly distributed cell population, which is highly desirable for both manual examination and automation. A conventional transmission light microscope (Labomed, CA, USA) was used to assess MN cells (MNC). The microscope was equipped with a 40x dry-lens plan objective, which possessed a numerical aperture of 0.65 and a field diameter of 450 µm at the level of the specimen. In the eyepiece of the microscope, a square grid, with dimensions of 1 × 1 cm^2^ and divided into 100 boxes measuring 1 mm^2^ each, was placed. Using this grid, a specific area that had been previously identified was analyzed, and a comprehensive assessment of 1000 cells per individual per sample was conducted based on the criteria described by Tolbert et al. [10]. The randomly selected slides were evaluated by two observers in a manner that prevented bias. This evaluation included examination of cells that showed MN, pyknotic nuclei, karyorrhexis, and karyolysis (Figures 1A, 1B).

Blinding

This was a double-blind study, in which the investigator who collected the samples and the oral pathologists were blinded to group allocation.

Reliability

Two-experienced oral pathologists (MS and PK) individually enumerated the numbers of MN, pyknosis, karyorrhexis, and karyolysis. The mean value was calculated based on the combined scores. The inter-observer reliability was determined to be 0.92, as measured using the kappa coefficient.

Statistical analysis

Data were curated and presented in Microsoft excel sheet and were analyzed using SPSS Statistics (version 22.0; IBM Corp., Armonk, NY, USA). The normal distribution of the data was studied using the Shapiro-Wilk test. As the data were found to be normally distributed, analysis of variance (ANOVA) was used to study differences between the groups, followed by post-hoc analysis using Tukey’s test. All levels of significance were set at P < 0.05.

## Results

The demographic characteristics of the study sample revealed that the mean age of the patients was 27.4±6.52 years, and both groups had an almost equal distribution of males and females, as shown in Table 1. 65% of the study population had a history of mild symptoms of COVID-19 and 35% had a history of moderate symptoms.

**Table 1:** Demographic characteristics of the study samples

Demographic variables	Control group	Case group
Gender (Male/Female)	11/9	12/8
Age (years) (Mean±SD)	28.1±6.69	27.4±6.52
Radiation exposure (last 6 months)	0	0
Antibiotic therapy (last one month)	0	0
Smoking habit	0	0
Oral mucosal lesion (last 6 months)	0	0

There were statistically significant differences in the number of MN, pyknosis, karyolysis, and karyorrhexis between cases and controls (p<0.01) at both sites. Considerably more genotoxic changes were observed in the mucosa of the tongue than in the buccal mucosa in both groups. COVID-19 patients displayed more genotoxic changes in the cells than the control group, as shown in Table 2.

**Table 2:** Comparative analysis of genotoxic markers (micronuclei, pyknosis, karyolysis, karyorrhexis) in cases and control using analysis of variance (ANOVA) test *p-value<0.01: Significant; **p-value<0.001: Moderately significant; ***p-value<0.0001: Highly significant

Group	N	Subgroup	Micronuclei (Mean±SD)	Pyknosis (Mean±SD)	Karyolysis (Mean±SD)	Karyorrhexis (Mean±SD)
Control (Group 1)	20	Buccal (Group 1a	42.45±14.04	154.25±16.93	170±26.45	25.95±9.38
		Tongue (Group 1b)	73.55±11.21	175.6±18.84	199.35±26.65	44.9±9.33
Cases (Group 2)	20	Buccal (Group 2a)	270±29.90	87.15±9.66	292.75±22.69	49.95±9.17
		Tongue (Group 2b)	293.9±23.09	120.9±10.77	321.95±29.97	58.4±15.21
P-value			0.001**	0.01*	0.0001***	0.001**

Post hoc analysis revealed statistically significant differences in the number of MN, pyknosis, and karyolysis between groups and subgroups (p0.05) (Table 3).

**Table 3:** Intra-group comparison of genotoxic markers using post-hoc Tukey’s test *p value<0.01: Significant; **p value<0.001: Moderately significant; NS: Not significant

Groups	Micronuclei	Pyknosis	Karyolysis	Karyorrhexis
Group 1a vs Group 1b	p<0.001**	p<0.001**	p<0.01*	p<0.001**
Group 1a vs Group 2a	p<0.001**	p<0.001**	p<0.001**	p<0.001**
Group 1a vs Group 2b	p<0.001**	p<0.001**	p<0.001**	p<0.001**
Group 1b vs Group 2a	p<0.001**	p<0.001**	p<0.001**	p<0.001**
Group 1b vs Group 2b	p<0.001**	p<0.001**	p<0.001**	p>0.05 (NS)
Group 2a vs Group 2b	p<0.01*	p<0.001**	p<0.01*	p>0.05 (NS)

## Discussion

The micronuclear assay is widely employed as a valuable biomarker in studies pertaining to genomic instability, genotoxic exposure, and initial biological response in human biomonitoring endeavors. The elicitation of MN is a highly efficient biomarker for diseases and phenomena linked to the elicitation of DNA damage [11]. Ceppi et al. reported a significant association between the frequency of MN in exfoliated buccal cells and the frequency of MN in lymphocytes. This association implies that genotoxic effects occurring within the bloodstream may affect buccal cells and can be detected. The same genetic factors and exposures influencing MN frequency in lymphocytes may also have a similar effect on buccal cells, possibly including a correlation between MN and cancer risk [12].

The aim of the present study was to assess the cytogenetic or genotoxic damage in buccal and tongue mucosal cells in COVID-19 patients and to determine whether SARS-CoV-2 affects the buccal and tongue mucosa differently. To date, no such study has been conducted. The present study revealed significant differences in the number of micronucleated, pyknotic, karyolytic, and karyorrhexis cells between COVID-19 patients and healthy individuals. Our findings are in accordance with the study conducted by Pinto et al. [7].

Infection with SARS-CoV-2 results in modifications of immune and metabolic responses within individuals, thereby generating an inflammatory milieu that greatly facilitates the occurrence of various infections. Hyper-inflammation is a hallmark of COVID-19 and is characterized by high levels of circulating proinflammatory cytokines, including interleukin-6 (IL-6), interferon-γ (IFN-γ), IL-1β, tumor necrosis factor (TNF), acute-phase reactants, and ferritin. However, understanding the intricate mechanisms underlying this phenomenon is challenging. SARS-CoV-2 specifically targets cellular entities that express ACE2 and TMPRSS2 [13]. The buccal mucosa and mucosa on the ventral surface of the tongue are lined by non-keratinized stratified squamous epithelium, where the suprabasal expression of ACE2 and TMPRSS2 is more, compared with the basal compartment [4]. Park et al. have reported similar findings [14].

Liu and colleagues discovered that cultured epithelial cells that carry the SARS-CoV-2 spike (S) protein have the ability to develop multinucleated syncytial cells when in contact with cells expressing ACE2, which serves as the receptor for the S protein. These fused cells manifested DNA damage and MN, which coexisted with the cytosolic DNA sensor cyclic GMP-AMP synthase (cGAS). This led to the activation of the adaptor protein stimulator of interferon genes (STING) and prompted the expression of genes responsible for type I IFNs and IFN-stimulated genes [15].

SARS-CoV-2 infection has the potential to elicit syncytia, a distinct cellular structure commonly found in human tumor tissues. The multinucleate syncytia often exhibited MN, which was closely linked to the activation of signaling pathways involved in DNA damage and cGAS-STING. This association provides a reasonable explanation for the abnormal activation of the immune system and the resulting extensive tissue damage [16].

In the current study, a higher incidence of genotoxic alterations was observed on the mucosa of the ventral surface of the tongue than on the buccal mucosa. This discovery is consistent with the findings of a study by Xu et al., who reported a greater abundance of ACE2 receptors on the tongue than on the buccal mucosa [17]. As mentioned in a previous study, the expression of ACE2 and TMPRSS2 is notably pronounced in the intermediate layer of squamous epithelia of the tongue [18].

Significant disparities were observed in cytotoxic modifications, such as pyknosis and karyolysis, within the cells of the tongue mucosa in COVID-19 patients when compared to individuals in good health. However, no significant differences were observed in karyorrhexis. Upon entry into the host cell, SARS-CoV-2 initiates RNA replication, protein expression, assembly of complete viral particles, and eventual release of virions. These processes occur concurrently with cell death and the subsequent release of cellular contents. These apoptotic or programmed cell death processes are characterized by specific morphological changes, including cell shrinkage (pyknosis), condensation and fragmentation of chromatin, fragmentation of the entire nucleus, cleavage of chromatin, and formation of apoptotic bodies (karyorrhexis) [18].

Karyolysis is the complete dissolution of chromatin in a dying cell due to enzymatic degradation by endonucleases. It usually occurs as a result of necrosis caused by external insults, such as viral infection [19]. The present study revealed significant karyolysis in the mucosa of the buccal mucosa and ventral surface of the tongue in COVID-19 patients, indicating necrosis and serious damage caused by the virus. This damage was more pronounced in the tongue than in the buccal mucosa. Our findings are in agreement with those of previous studies in which SARS-CoV-2 was associated with apoptosis and necrosis of host cells [19,20].

Clinical significance

Genotoxic or cytogenetic alterations in cellular entities caused by viral etiology act as harbors of carcinoma progression in subsequent times. Consequently, COVID-19 patients should undergo periodic surveillance in order to detect emerging carcinomas, primarily in the oral cavity, starting with the tongue and progressing to the buccal mucosa. Such advancements are more likely to manifest in COVID-19 individuals who engage in habits such as smoking, tobacco consumption, prior exposure to radiation, and the presence of systemic ailments.

Limitations

The current investigation was conducted with a limited sample size, and the analysis did not include an assessment of sexual disparities. The investigation excluded Individuals afflicted with severe and critical cases were COVID-19. Subsequent prospective investigations are warranted to assess cytogenetic alterations in cells at various locations within the oral mucosa of patients afflicted with mild, moderate, severe, and critical forms of COVID-19.

## Conclusions

The current study proposes that SARS-CoV-2 can initiate genetic variations and cellular toxicity within oral cells. These effects were more pronounced on the tongue mucosa than on the buccal mucosa. However, additional investigations are necessary to achieve a more comprehensive understanding of whether SARS-CoV-2 stimulates mutagenesis, alongside cytotoxicity, in other areas, such as the gingiva and nasal cells. This valuable knowledge will considerably enhance our understanding of the disease and contribute to the development and validation of oral diagnostics for COVID-19.
